# Detection of copy number variations and their effects in Chinese bulls

**DOI:** 10.1186/1471-2164-15-480

**Published:** 2014-06-17

**Authors:** Liangzhi Zhang, Shangang Jia, Mingjuan Yang, Yao Xu, Congjun Li, Jiajie Sun, Yongzhen Huang, Xianyong Lan, Chuzhao Lei, Yang Zhou, Chunlei Zhang, Xin Zhao, Hong Chen

**Affiliations:** College of Animal Science and Technology, Northwest A & F University, Shaanxi Key Laboratory of Molecular Biology for Agriculture, Yangling, Shaanxi China; United States Department of Agriculture-Agricultural Research Service, Bovine Functional Genomics Laboratory, Beltsville, Maryland USA; Institutes of Cellular and Molecular Biology, Jiangsu Normal University, Xuzhou, Jiangsu China; Red Sea Research Center, King Abdullah University of Science and Technology (KAUST), Thuwal, Saudi Arabia

**Keywords:** Copy number variations, *Bos taurus*, *Bos grunniens*, *Bubalus bubalis*, Gene expression

## Abstract

**Background:**

Copy number variations (CNVs) are a main source of genomic structural variations underlying animal evolution and production traits. Here, with one pure-blooded Angus bull as reference, we describe a genome-wide analysis of CNVs based on comparative genomic hybridization arrays in 29 Chinese domesticated bulls and examined their effects on gene expression and cattle growth traits.

**Results:**

We identified 486 copy number variable regions (CNVRs), covering 2.45% of the bovine genome, in 24 taurine (*Bos taurus*), together with 161 ones in 2 yaks (*Bos grunniens*) and 163 ones in 3 buffaloes (*Bubalus bubalis*). Totally, we discovered 605 integrated CNVRs, with more “loss” events than both “gain” and “both” ones, and clearly clustered them into three cattle groups. Interestingly, we confirmed their uneven distributions across chromosomes, and the differences of mitochondrion DNA copy number (gain: taurine, loss: yak & buffalo). Furthermore, we confirmed approximately 41.8% (253/605) and 70.6% (427/605) CNVRs span cattle genes and quantitative trait loci (QTLs), respectively. Finally, we confirmed 6 CNVRs in 9 chosen ones by using quantitative PCR, and further demonstrated that CNVR22 had significantly negative effects on expression of *PLA2G2D* gene, and both CNVR22 and CNVR310 were associated with body measurements in Chinese cattle, suggesting their key effects on gene expression and cattle traits.

**Conclusions:**

The results advanced our understanding of CNV as an important genomic structural variation in taurine, yak and buffalo. This study provides a highly valuable resource for Chinese cattle’s evolution and breeding researches.

**Electronic supplementary material:**

The online version of this article (doi:10.1186/1471-2164-15-480) contains supplementary material, which is available to authorized users.

## Background

Copy number variation (CNV), as a form of widespread genomic structural variations, has been reported in many model organisms, such as primates and rodents [1–5]. Compared with single nucleotide polymorphisms (SNPs), CNVs seem to have a stronger impact on phenotype and are shown to have effects on changes in gene expression levels [6], which can be explained by disruption of gene dosage, unmasking of recessive alleles, and loss of regulatory elements or regulatory polymorphisms [7, 8]. Several recent publications have reviewed the effects of CNVs on gene expression and human diseases [9–11]. In addition, CNV provides materials and mechanisms for creating new genes [12].

Given the importance of CNVs and their high rates of mutation, interest in CNV detection has extended to domesticated animals, including dogs [13], pigs [14], goats [15], horse [16], and sheep [17]. Similarly, CNV and copy number variable regions (CNVRs) have been a hot-spot in cattle genomic variation researches, which may be associated with, or affect, cattle’s health and production traits under recent selection. Previous studies have produced several CNV datasets on cattle [18–26]. Some are focused on one single breed by using SNP array: such as, *Bos taurus* coreanae (855 CNVs and 368 CNVRs in 265 individuals [20]) and Chinese Holstein cattle (367 CNVRs in 96 individuals [23] and 99 CNVRs in 2,047 individuals [24]). Other CNV evidences for multiple breeds are also shown on SNP array. For example, Matukumalli *et al*. identified 79 candidate deletions by using an earlier version of BovineSNP50 assay [27], and Hou *et al*. found 682 candidate CNVRs in 21 modern cattle breeds and 6 out-groups [21]. At the same time, more studies are conducted on microarray-based comparative genomic hybridization (array CGH): 177 high-confidence CNVRs in 17 breeds [18], and 304 CNVRs in 4 breeds [19]. Most recently, the next-generation sequencing was also used to detect CNVR with more power [22, 25, 26]. And in these studies, the researchers focus more on the detection of CNV in different breeds.

Up to this date, few studies have confirmed the genome-wide presence of CNVs in Chinese native cattle breeds. Compared to the previous CNV investigations mostly focusing on CNV detection, here we selected 15 breeds in three main bovine groups in China (twelve *B. taurus*, one *Bos grunniens*, and two *Bubalus bubalis* ones) to conduct a genome-wide CNV analysis and further examined their effects on gene expression and growth traits of cattle. Overall, we got started with genome-wide CNV screening of three cattle groups, and further associated them with cattle gene expression and body measurements, which provides novel insights into understanding the role of CNV in genomic variation studies.

## Methods

### Sample collection

For CGH analysis, we collected blood samples all over China in 15 bovine breeds or populations: twelve *B. taurus* breeds (taurine): Anxi, Bohaihei, Chinese Holstein, Jiaxian, Jinnan, Leiqiong, Luxi, Mongolia, Nanyang, Qinchuan, Wannan and Zaosheng; one *B. grunniens* (yak): Tianzhu White yak; and two *B. bubalis* ones (buffalo): Swamp buffalo and River buffalo (Additional file 1: Table S1).

Diverse tissues of fetal, calf (including heart, liver, spleen, lung, kidney, and muscle) and adult (including heart, liver, spleen, lung, kidney, stomach, intestine, muscle, and adipose) in Qinchuan breed were collected in the slaughter house for gene expression analysis (Additional file 1: Table S1). Blood samples of Nanyang (NY, N = 43), Jiaxian (JX, N = 39) and Qinchuan (QC, N = 47) were collected with body measurements (older than 2 years old), including body height, body length, heart girth, hucklebone width, and body weight for association analysis. All our sample collection was carried out in strict accordance with the ethical guidelines approved by the Animal Care Commission of College of Animal Science and Technology, Northwest A & F University.

Genomic DNA was extracted and purified from whole blood following standard procedures [28] and quantified by spectrophotometry and agarose gel electrophoresis. Total RNA was isolated from flash-frozen tissues. First-strand cDNA was synthesized from 500 ng of total RNA with the Prime Script RT Reagent Kit (TaKaRa, Dalian, China) according to the manufacturer’s instructions.

### Array CGH platform

We quantified copy number by hybridizing DNA to Nimblegen3x720K CGH array (http://www.nimblegen.com), which provided an evenly distributed coverage of ~720,000 oligonucleotide probes (mean probe spacing: 3,364 bp, array No. GPL17177). The probes of 50–75 bp in length were designed with similar melting temperatures based on Btau_4.0 genome assembly [29].

We chose one pure-blooded Angus bull as the reference. DNA labelling, hybridization, washing, array scanning, and array imaging were carried out according to the previously described [30]. Briefly, pairs of genomic DNA (1 μg) were labeled with fluorescent dyes Cy3 (test sample) or Cy5 (reference), and were co-hybridized on hybridization platform. The arrays were scanned and fluorescent intensity raw data was extracted. The initial data analysis (normalization and segmentation) was performed on NimbleScan v2.4 software with segMNT algorithm (Capital Bio Corporation, Beijing, China). We used an updated version of the previously described method to do CNV calling, ie., determining copy number gains and losses by changes in log2 signal intensity [31]. The segment, with mean log2 ratio ≥ |0.5| and at least 5 consecutive probes covered, was defined as a CNV. CNVRs in one group were determined by aggregating overlapped CNVs of all samples [32].

Cattle gene annotations were downloaded from the UCSC genome browser (http://hgdownload.cse.ucsc.edu/downloads.html#cow), and cattle quantitative trait loci (QTLs) [33] were from the Animal QTL database (http://www.animalgenome.org/cgi-bin/QTLdb/BT/index). The genome positions were converted among genome assemblies of Btau_4.0, Btau_4.6, and Btau_4.6.1 by using the UCSC binary software LiftOver. We wrote Perl scripts to search for gene content and quantitative trait loci (QTLs) inside CNVRs, and determined a positive gene/QTL by > 50% overlap. Gene ontology (GO) identifiers were retrieved with Refgene IDs in R package biomaRt and plotted by the web histogram tool WEGO (http://wego.genomics.org.cn/cgi-bin/wego/index.pl) [34]. CNVR chromosome plotting, clustering analysis, nonmetric multidimensional scaling (NMDS), GO identifier retrieving, and indicator species analysis (ISA) were performed by using R packages of ggbio, pvclust, vegan, biomaRt, and indicspecies, repectively. Principal component analysis (PCA) was performed in STAMP v2.02 [35].

### Data access

Raw array CGH data in this study has been deposited in NCBI GEO database under accession number of GSE47086.

### Quantitative PCR (qPCR)

We performed a qPCR analysis to validate copy number changes detected by array CGH based on the relative comparative cycle threshold (CT) method. Primers (Additional file 1: Table S2) were designed by using Beacon Designer™ (PREMIER Biosoft, USA). PCR reaction was done in a volume of 20 μL containing 20 ng of genomic DNA, 0.4 μM of each primer, and SYBR Premix Ex *Taq*™ II reagents (TaKaRa Biotechnology, Dalian, China). Analysis of resultant crossing thresholds (Ct) was performed based on the ΔΔCt method [36], and ΔΔCt values were determined by comparing test samples and Angus reference (two-copy states) with *BTF3* gene as internal control. Finally, the relative copy number for each sample was calculated as 2^-ΔΔCt^.

We selected two qPCR-confirmed CNVR22 and CNVR310 for further analysis. First, we used the primers (Additional file 1: Table S2) to determine CNVR types (gain, loss or normal) in 30 individuals against the Angus reference. Then we selected checked individuals (20 samples for CNVR22, 3 of gain, 2 of loss and 15 of normal; 15 samples for CNVR310, 4 of loss and 11 of normal) for gene expression analysis of *PLA2G2D* in CNVR22 and *MYH3* in CNVR310 on the CFX-96 Real-Time PCR Detection System (Bio-Rad, Hercules, CA, USA).

Two primer pairs were used for expression detection of *PLA2G2D* and *MYH3*: *PLA2*-F, 5′-GACATACTGGACCTGAAC-3′; *PLA2*-R, 5′- AGCCATAGTGTGAATAGAAG-3′; *MYH3*-F, 5′-AGTCGTCAGTTGGAGGAA-3′; *MYH3*-R, 5′-GCTCTTCTATTTGCTGGGTAA-3′. *GAPDH* gene was used for normalization. The reaction was performed in a volume of 25 μL, containing 12.5 μL SYBR Premix Ex *Taq* II, 1 μL of each primer (10 μM), 2 μL cDNA (2.5 ng/μL), and 9.5 μL H_2_O. The relative fold change was calculated using 2^-ΔΔCt^[36]. Mean expression levels and standard deviations were obtained by repeating three independent experiments.

### Association analysis between CNVR types and growth traits

We evaluated all kinds of factors, and selected three major ones of farm, genotype and breed to build a reduced adjusted linear model:1

where Y_*ijk*_ is trait measurement, μ is overall population mean, F_*i*_ is farm, G_*j*_ is genotype effect, B_*k*_ is breed, and E_*ijk*_ is random error.

We used the least-squares means (LSM) to estimate the association between CNVR types and body measurements in SPSS software [37].

## Results and discussion

### CNVRs in cattle groups

**Figure 1:**
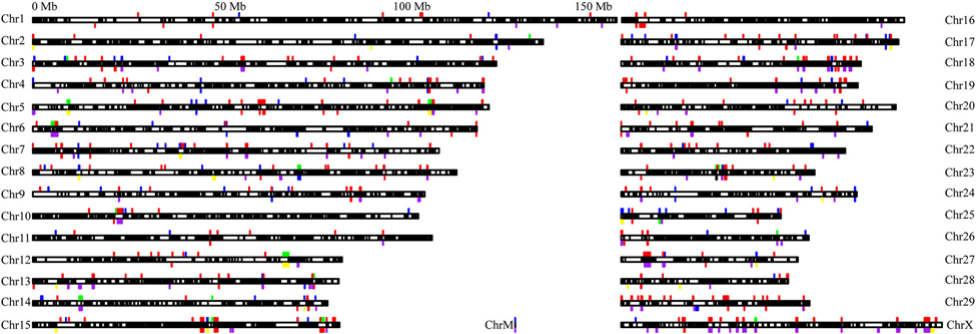
**Genomic distribution of CNVRs in Chinese bulls.** 605 CNVRs (~3.04% of the bovine genome) in 29 bulls are distributed across chromosomes, in which the above are for taurine (green: both, red: loss, dark blue: gain), while below are the CNVRs for yak (the same colors to that in taurine) and buffalo (black: both, purple: loss, yellow: gain). Refgenes from UCSC genome browser are shown inside the chromosomes in black.

In taurine, we identified 370 CNVRs covering the region of 47 Mb on the placed chromosomes (1.78% of the placed chromosome in Btau_4.0), together with 116 CNVRs on ChrUnAll (unassigned sequence contigs). All 486 CNVRs correspond to 2.45% of the bovine genome (71.5/2,918.1 Mb), which consist of 329 loss, 113 gain and 44 both (both: loss and gain within one CNVR) events (Table 1, Figure 1, and Additional file 1: Table S3). Loss events are approximately 3-fold more than gain ones, while both ones are much longer than the others on average. Furthermore, 96 CNVRs are only found in one individual, and 390 CNVRs are shared in two or more ones, among which 51 multiple events have a frequency of ≥ 0.5. In yak/buffalo groups, we identified 161/163 candidate CNVRs, which consist of 123/131 loss, 34/31 gain, and 4/1 both events. Duplications provide additional copies of genes, and this kind of redundancy can allow more flexibilities of gene loss by selective pressure. Mutation and selection can result in functional changes introducing a new function or specialization of old functions [38, 39]. Thus, gain events might be the first step for extra genetic material during cattle breed formation, and their followed isolated genome CNV evolution and adaption may be a potent evolutionary force for more loss events.

**Table 1:** **CNVR summary for Chinese bulls**

	Sample	CNVRs	Avg	Unique	Gain	Loss	Both	%*
Taurine	24	486	20.3	96	113	329	44	2.45%
Yak	2	161	80.5	68	34	123	4	1.25%
Buffalo	3	163	54.3	45	31	131	1	1.44%
Merged	29	605	20.9	95	126	422	57	3.04%

*percentage of total CNVR length in the genome assembly of Btau_4.0.

“Avg” is for CNVRs per sample.

**Figure 2:**
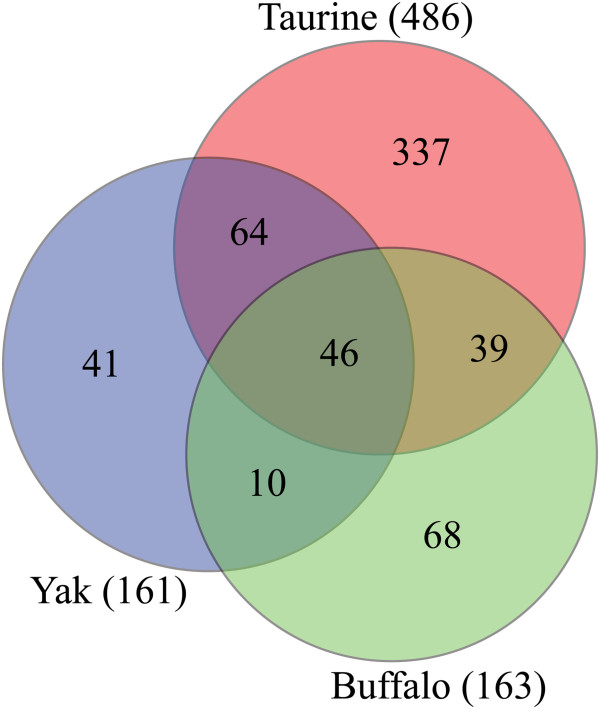
**Venn diagram of CNVRs in three groups (taurine, yak, and buffalo).** CNVRs are overlapped among taurine, yak, and buffalo in the specific area.

On combining three groups, we identified 605 CNVRs totally, 3.04% of the bovine genome, among which 110 ones in yak (110/161, 68.3%) and 85 ones in buffalo (85/163, 52.1%) are shared by taurine (22.6% and 17.5%, respectively), and about 46 CNVRs are overlapped in all three groups (Figure 2). We then compared 31 shared CNVRs on the placed chromosomes to those in previous studies (Additional file 1: Table S4), and confirmed them all, except for CNVR124 and CNVR127, which indicated their reliability. It is notable that CNVR frequencies in different studies are shown diverse, and more shared CNVRs are detected based on CGH and re-sequencing methodologies than that of SNP-array-based studies (Table 2). And cattle breeds, which may have experienced different selection pressures, contribute a lot to CNVR differences as well. It concludes that samples and platform may have the greatest effects on CNV detection.

**Table 2:** **CNVRs shared by this study and other studies on cattle CNV**

	Study	Breeds	Samples	Shared CNVRs*	Total CNVRs
Re-sequencing	Bickhart *et al*. [22]	3	6	24	1265
	Zhan *et al*. [26]	1	1	12	521
	Stothard *et al*. [25]	2	2	10	790
CGH array	This study	14	29	31	605
	Liu *et al*. [18]	17	90	23	177
	Fadista *et al*. [19]	4	20	12	304
SNP array	Hou *et al*. [21]	21	539	10	682
	Bae *et al*. [20]	1	265	1	855
	Jiang *et al*. [24]	1	2047	2	99
	Jiang *et al*. [23]	1	96	3	367

*Shared CNVRs do not contain the ones on ChrUnAll.

We performed an integrated hybridization on the three bovines in one CGH platform, and gained an advantage of parallel comparison, regardless of how different their genomes are. Our array probes were designed based on genome assembly Btau_4.0 (a Hereford cow) [40]. Yak genome had been released after our CGH experiment was done, and shown very similar to that of cow [41, 42]. Up to date, the complete genome of buffalo is not placed well in NCBI, and only unplaced sequences are ready to download. All our CNVRs in yak and buffalo are from the orthologous regions of cow genome, and our 46 shared CNVRs are supposed to be from the common orthologs of the three bovine genomes. However, their genome positions in yak and buffalo may be different and should be considered cautiously. And the yak- and buffalo-specific regions are not covered by our probes, and out of our scope. It is noted that the bovine CGH array had also been used to scan CNV of goat and sheep [15, 17].

In addition, we designed five probes covering the mitochondrial region. Although there is only one CNVR in ChrM in three groups, we found a difference between taurine (gain) and yak (loss) & buffalo (loss) (Additional file 1: Table S5). The mitochondrion DNA copy number varies referring to energetic metabolism among cell types [43] and fertility between oocytes and sperm [44]. Multiple copies of mtDNA in the same mitochondrion are directly associated with the amount of ATP synthesized [45, 46]. Our finding on mtDNA copy number difference suggests possible low energetic metabolism in yak and buffalo’s blood, and more studies are necessary to understand the role of mitochondrial copy number in cattle’s traits and performances.

We had discovered CNVRs’ distribution preferences across chromosomes. Just like the previous results [18, 21], cattle CNVs are distributed in a non-random way in this study, and their contents vary across chromosomes. The proportion of any known chromosome susceptible to CNVRs ranges from 0.3 to 4.07% (Figure 1), although ChrUnAll shows the strongest enrichment of CNVRs (8.21%), probably due to highly repetitive sequences in these unplaced contigs. Except for ChrM, we got strong CNV-enriched chromosomes of 27, 18, 15, 29, 5, × and 23 (>2.13% on average), which might have been shaped by local Chinese bulls, compared with other results [18, 21].

### Clustered cattle CNVRs

Selection has also been shown to shape the architecture of segmental duplications during human genome evolution [47], and studying CNVs’ evolution may help us reveal the genomic alteration and environmental driving impact. The cluster analysis of CNVR in cattle and pig had evidenced that CNVR loci are consistent with the breed divergence and history [14, 18]. So we performed a clustering analysis of CNVRs on all individuals, which revealed remarkable profiles among groups. First, the three groups of taurine, yak, and buffalo are clearly divided (Additional file 2: Figure S1). Second, individuals in one single breed are easily clustered closely. The clustering results simply showed a phylogeny, while the principal component analysis (PCA) results showed their detailed relations. The PCA plotted samples into three groups (Additional file 2: Figure S2A). Similarly, we investigated the impact of groups on CNVRs structure by using nonmetric multidimensional scaling (NMDS), which is to visualize the interrelationships among a complex dataset and level of similarity of individuals, and generally grouped samples into taurine, yak, and buffalo (Additional file 2: Figure S2B). The results supported the hypothesis that genome structure variations, especially CNVs, may be raised by isolated evolutions and shaped by breed formation and adaptation [18].

To highlight potential evolutionary contributions of CNVs to Chinese major cattle breeds’ formation and adaptation, we identified 130 CNVRs which are abundant statistically in three groups (Additional file 1: Table S3) by using indicator species analysis (ISA) [48]. Compared to 46 shared CNVRs from the orthologs, the biased ones reflect their unique genomic backgrounds, which suggest a potential variance of the three bovine genomes. Our parallel comparisons based on one single CGH platform are reliable, which overcome the shortcoming that it is difficult to compare the datasets by different technologies and methods.

### Gene content and quantitative trait loci (QTLs) in CNVRs

Totally 253 CNVRs encompass 716 genes, which are shown with refGene ID and gene name (Additional file 1: Table S3). In order to determine biological functions of copy number variable genes, a gene ontology (GO) analysis annotated 647 out of 716 genes in three main GO categories: cellular component, molecular function and biological process (Additional file 2: Figure S3). As shown in the GO map, genes in all categories were mainly involved in eight ones, including cell/cell part (mainly intracellular), organelle (mainly intracellular organelle part), binding (mainly protein binding), catalytic activity (mainly hydrolase activity and transferase activity), metabolic process (mainly, primary, macromolecule, cellular, and nitrogen compound metabolic process), cellular process (mainly cellular metabolic process), pigmentation (mainly regulation of cellular process), and biological regulation (mainly regulation of biological process). This set of copy number variable genes possesses a wide spectrum of molecular functions, and provides a rich resource for hypotheses on their genetic basis of phenotypic variation within and among breeds. Many cattle specific genes were also found in our CNVRs, such as C-type lysozymes, *BSP30A*, interferon tau subfamilies, *WC1*, and *ITLN1*[49]. Moreover, *EDA* gene, which has been reported a deletion and responsible for hypotrichosis and dental defects in cattle [50], and *SLC4A2* gene, which is a copy number variable gene and association with osteopetrosis, are both confirmed in our study [51].

We also downloaded 8035 cattle QTLs from Animal QTLs database, and searched for potential QTLs which reside inside 477 CNVRs on placed chromosomes (Additional file 1: Table S3). There are 89.5% (427/477 on placed chromosomes) of CNVRs overlapped with 1186 QTLs. The QTLs in multiple CNVRs are associated with exterior (20 QTLs), health (107 QTLs), meat and carcass (275 QTLs), milk (254 QTLs), production (302 QTLs) and reproduction (228 QTLs). The results are in accordance with Chinese breeding history.

### CNVR confirmation and effects on gene expression

To evaluate the accuracy of copy number assignments, quantitative real time-PCR was used as described previously [18]. We selected nine detected CNVR, including loss, gain, and both types, whose frequencies range from 4.17% to 37.50%. The selected CNVRs were all overlapped with genes and QTLs of meat, carcass and production (Additional file 1: Table S2). Totally 14 pairs of primers were used, one or two pairs to cover one selected CNVR. The results showed that out of 14 qPCR assays, 10 ones (71%) confirm the predictions by array CGH. False-positive identification is common in CNV detection, and there is always to some extent < 1 of confirming rate [18, 19, 21]. In fact, CNVRs were of complex structure, and qPCR can only target a small portion, which does not reflect their complete characteristics. And the boundaries of CNVR by arrays are indistinct. Notably the average size of 9 confirmed CNVRs is 63.40 kb, much smaller than those of three unconfirmed ones (72.18 kb). Certain number of samples are chosen randomly as negative control for the reliability of results [14].

**Figure 3:**
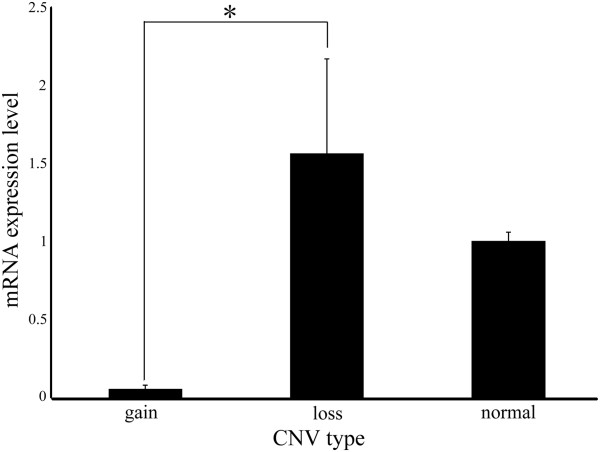
**Relative mRNA expression level of** ***PLA2G2D*** **in CNVR22 in adipose tissues.** Relative *PLA2G2D* mRNA expression levels in adipose tissues of 20 selected Qinchuan cattle individuals (3 gain, 2 loss, and 15 normal ones) were analyzed by qPCR, and normalized against that of *GAPDH*. CNV types were determined against the Angus reference, and the normal type means the same to the reference. Three independent experiments were repeated for reliability. An asterisk denotes a significant difference by t-test (*P* < 0.05).

In rats, only 44% genes in CNVRs are differentially expressed [52]. We selected two genes (*MYH3* and *PLA2G2D*) for detailed exploration of expression levels, because both of them might have effects on the performances of cattle. *PLA2G2D* is an innate immunity gene, and thought to play a role in gonadotropin-releasing hormone and MARK signaling [53], which had also been identified inside a CNVR of Black Angus by re-sequencing method [25]; most recently, it was confirmed that the copy numbers of *PLA2G2D* gene were associated with the index of total merit in Holstein bulls [54]. The findings were very important because it was the direct evidence of complex traits of livestock which may be modulated in part by CNVs. And *MYH3* is expressed mainly in embryo and muscle [55], and its mutation caused the Freeman-Sheldon and Sheldon-Hall syndrome [56]. In cattle, the SNP in *MYH3* was also associated with the growth and carcass traits in Chinese Qinchuan cattle [57]. So, we firstly examined the expression profiles of both genes in Qinchuan cattle. The results showed that mRNA of *PLA2G2D* was mainly expressed in spleen, intestine, adipose, and lung (Additional file 2: Figure S4A), while *MYH3* mRNA was primarily expressed in fetal muscle, and liver, spleen and muscle of calf (Additional file 2: Figure S4B). Secondly, considering both tissue importance in growth traits and mRNA expression, we selected 20 samples of adult adipose tissues (3 of gain, 2 of loss and 15 of normal) and 15 ones of calf muscle tissues (4 of loss and 11 of normal) to analyze CNVR’s effects on mRNA of *PLA2G2D* and *MYH3*, respectively. The results revealed a significant negative correlation between mRNA levels of *PLA2G2D* and CNVR22 (Figure 3). The correlation could be due to position effect of CNVR, and a regulatory sequence of *PLA2G2D* may exactly reside in CNVR22 [58]. Interestingly, the regulatory sequence may be a transcriptional upper repressor and suppressed *PLA2G2D* mRNA expression. However, we have not seen any evidence of correlation between *MYH3* transcript expression and CNVR310. It is well known that the break point definition of CNVRs by arrays is equivocal and only 44.79% of *MYH3* gene is overlapped with CNVR310. So it is possible that *MYH3* or its regulatory motif was not covered by CNVR310. It is also worth emphasizing that the dosage compensation, lack of regulatory elements in the duplicated copy, differences in the chromatin environment and many other factors might keep mRNA levels stable [59].

### CNVs’ association with growth traits of cattle

CNVs may affect phenotype by altering transcriptional level of genes within or adjacent to CNVR and subsequently alter translation levels [60, 61]. The association between the CNVs and production traits of economic interest had been reported. In swine, several copy number variable genes were identified as candidate genes for phenotypes related to carcass length, backfat thickness, abdominal fat weight, length of scapular, intermuscle fat content of logissimus muscle, body weight at 240 day, and fatness [62]. In cattle, the direct evidence of close associations of CNVR#456 with index of total merit and genetic evaluations for protein production, fat production, and herd life in Holstein had been identified [63]. We had demonstrated that the genes inside CNVRs might be expressed differently, and many CNVRs also overlapped with QTLs which are associated with cattle performance. So we believed that CNVRs should be potentially associated with cattle body measurements.

Here we collected 129 Chinese cattle samples in Qinchuan, Jiaxian and Nanyang breeds (Additional file 1: Table S6), and evaluated the associations between CNV types and growth traits (including body height, body length, heart girth, hucklebone width, and body weight) in Equation 1. The results indicate that heart girth and body length are significantly associated with CNV types in CNVR22 (Table 3). Individuals of loss type have larger heart girth and hucklebone width (*P* < 0.05). The location of CNVR22 on chromosome 2 exactly fell into QTLs with various functions, including QTLs 10670 and 1390 for production (body weight), QTLs 5812 (palmitoleic acid content) and 11725 (marbling score) for meat and carcass [64, 65] in beef cattle (Additional file 1: Table S3). Furthermore, *PLA2G2D* gene was overlapped with CNVR22. Cattle of loss type in CNVR22 with higher *PLA2G2D* expression maybe finally gain more fat deposition. In addition, association between CNV of *PLA2G2D* gene and index of total merit had been reported in Holstein [54].

**Table 3:** **Association analysis of CNVR22 with body measurements**

	N	Body height*	Body length*	Heart girth*	Hucklebone width*	Body weight*
Gain	28	126.7 ± 5.3	133.9 ± 9.5^b^	169.9 ± 10.6^b^	23.2 ± 2.3	354.7 ± 48.0
Loss	14	125.5 ± 6.0	145.2 ± 7.8^a^	176.0 ± 12.0^a^	20.4 ± 6.1	385.1 ± 62.9
Normal	87	126.9 ± 6.2	139.9 ± 10.1^a^	174.5 ± 10.0^a^	21.3 ± 4.6	368.5 ± 59.7
Total	129	126.7 ± 6.0	139.2 ± 10.2	173.7 ± 10.5	21.7 ± 4.4	367.3 ± 57.9

*LSM ± SE, “*LSM*” for least squares mean, “*SE*” for standard error. “*N*” indicates sample number.

^a, b^Means with different superscripts were significantly different (*P* < 0.05).

On the other hand, we also found that CNVR310 is significantly associated with heart girth (*P* < 0.05), probably due to the fact that CNVR310 was overlapped with the QTLs for production (QTLs 11079 and 5297) and meat and carcass (QTLs 10021, 12174, 1395, and 22873) in beef cattle (Additional file 1: Tables S3 and S7). Regardless of one single gene’s contributions, we had a direct look at the effects of selected CNVRs on cattle body performances, which might be caused by a group of genes. It took gene population effects into consideration, rather than focusing on only one single trait-related gene. The association between CNVs and traits is inspiring, but larger population may be needed to validate it.

## Conclusions

We have performed a comprehensive genomic analysis of CNVs based on CGH arrays in Chinese cattle, and a detailed functional investigation for CNVRs’ effects on both gene expression and cattle body measurements. We identified 486 CNVRs in *B. taurus*, which covered 2.45% of the bovine genome, together with 161 and 163 CNVRs in *B. grunnies* and *B. bubalis*. Furthermore, we confirmed that CNVR22 had significantly negative effects on both *PLA2G2D* gene expression and cattle body measurements, while CNVR310 showed a significant negative association with heart girth. Our results generated a valuable genome-wide variation resource for Chinese cattle genomic researches, and provided a novel insight into understanding the association between animal complex traits and CNVRs during their adapting to local geographical environment and domesticated needs from human society.

## Electronic supplementary material

Additional file 1: Table S1.1: The breeds or populations of Chinese cattle used in the CGH arrays. **Table S1.2.** The breeds or populations of Chinese cattle used in other experiments. **Table S2.** Primer sequences and results for qPCR. **Table S3.** CNVRs in Chinese bulls. **Table S4.** Shared CNVRs by this study and other studies. **Table S5.** CNVR in ChrM in Chinese bulls. **Table S6.** CNV types of individuals on CNVR22 and CNVR310 in three Chinese breeds. **Table S7.** Association analysis of CNVR310 with body measurements. (XLSX 136 KB)

Additional file 2: Figure S1: Clustering results of CNVRs. **Figure S2.** PCA and NMDS of CNVRs on Chinese bulls. **Figure S3.** Gene ontology (GO) annotations for genes covered by CNVRs. **Figure S4.** Expression pattern analysis of *PLA2G2D* gene and *MYH3* gene. (PDF 737 KB)

## Abbreviations

CNV: Copy number variations
CNVR: Copy number variable region
CGH: Comparative genomic hybridization
GO: Gene ontology
MYH3: Myosin-3, *B. taurus* myosin, heavy chain 3
ISA: Indicator species analysis
PLA2G2D: *B. taurus* calcium-dependent phospholipase A2
NMDS: Nonmetric multidimensional scaling
PCA: Principal component analysis
qPCR: Quantitative real time PCR
QTLs: Quantitative trait loci
SNP: Single nucleotide polymorphism.
