# Supplementary material for: Detection of copy number variations and their effects in Chinese bulls
Source: BMC Genomics. 2014 Jun 17;15(1):480. doi: 10.1186/1471-2164-15-480 (PMC4073501; doi:10.1186/1471-2164-15-480)
Supplement: Supplementary file 2 — Additional file 2: Figure S1: Clustering results of CNVRs. Figure S2. PCA and NMDS of CNVRs on Chinese bulls. Figure S3. Gene ontology (GO) annotations for genes covered by CNVRs. Figure S4. Expression pattern analysis of PLA2G2D gene and MYH3 gene. (PDF 737 KB) [file 12864_2013_6164_MOESM2_ESM.pdf]

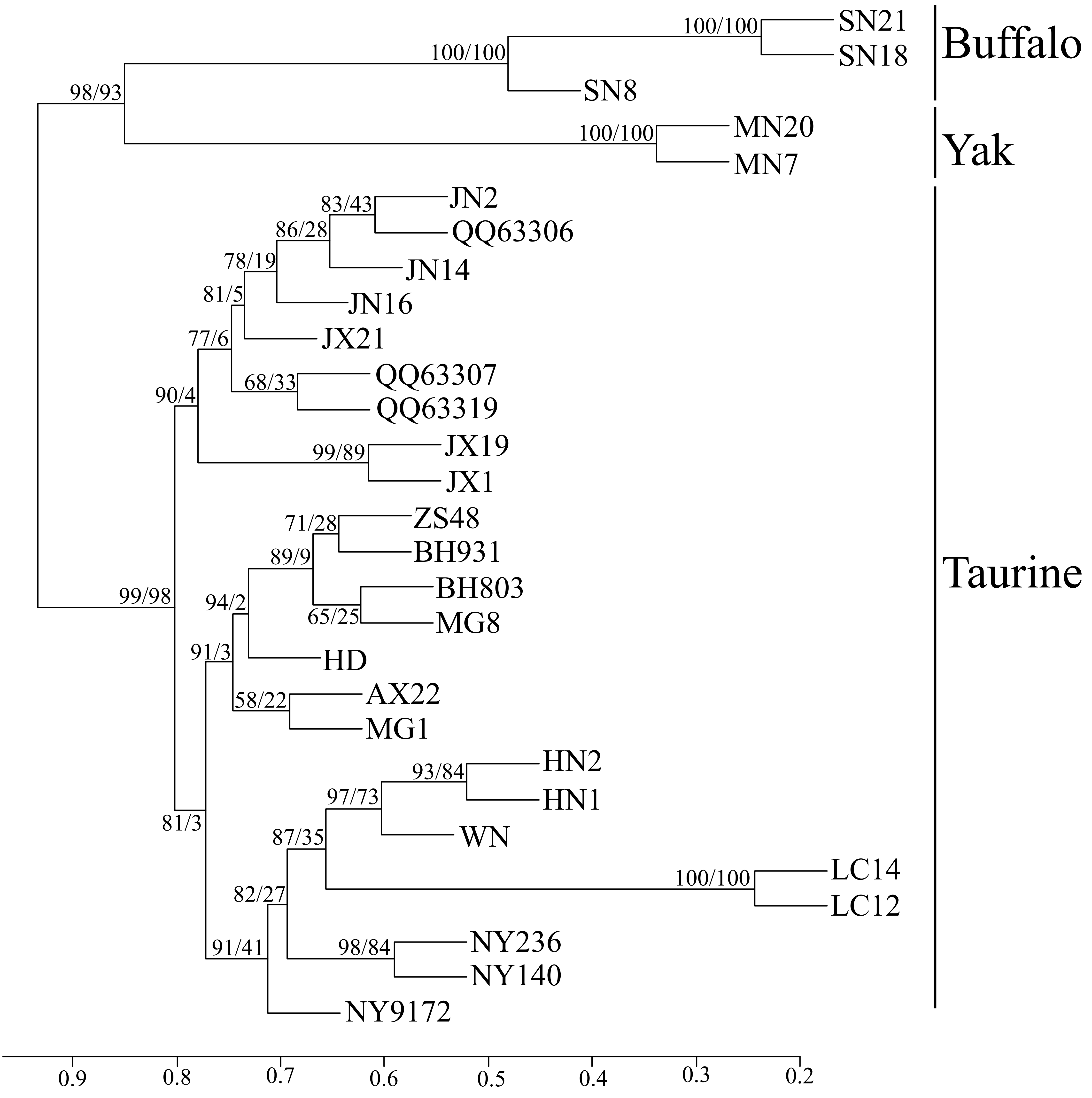

Figure S1 Clustering results of CNVRs. Chinese bulls are grouped into taurine, yak, and buffalo. The hierarchical clustering of samples by CNVRs was done in R package pvclust with a cluster method of "average" and distance measure of "correlation", and "approximately unbiased p"/"bootstrap probability value" are shown on the branches.

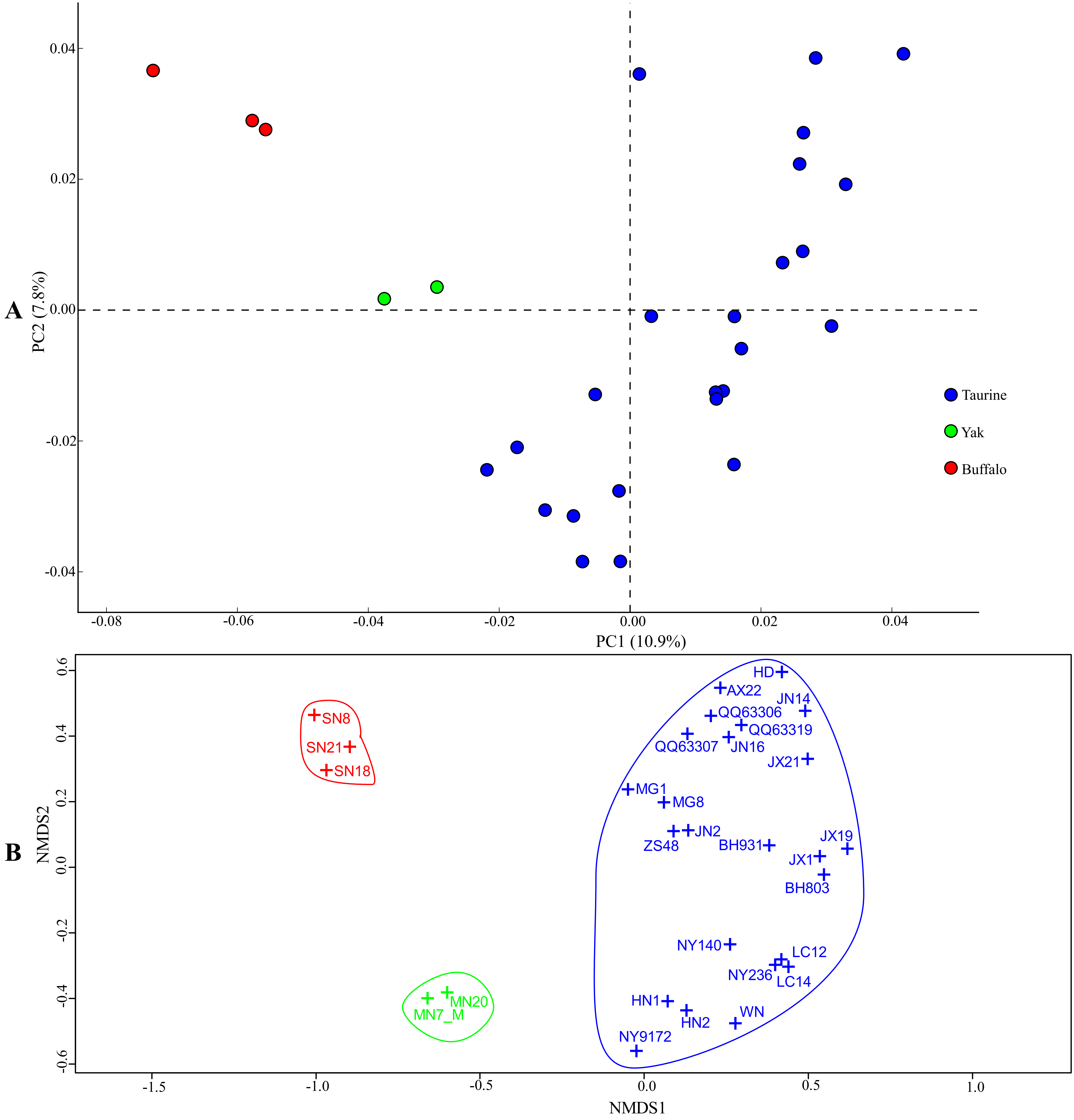

Figure S2 PCA and NMDS of CNVRs on Chinese bulls. A. PCA of CNVRs on all Chinese bulls. B. NMDS of CNVRs on all Chinese bulls. Taurine, blue; Yak, green; Buffalo, red.

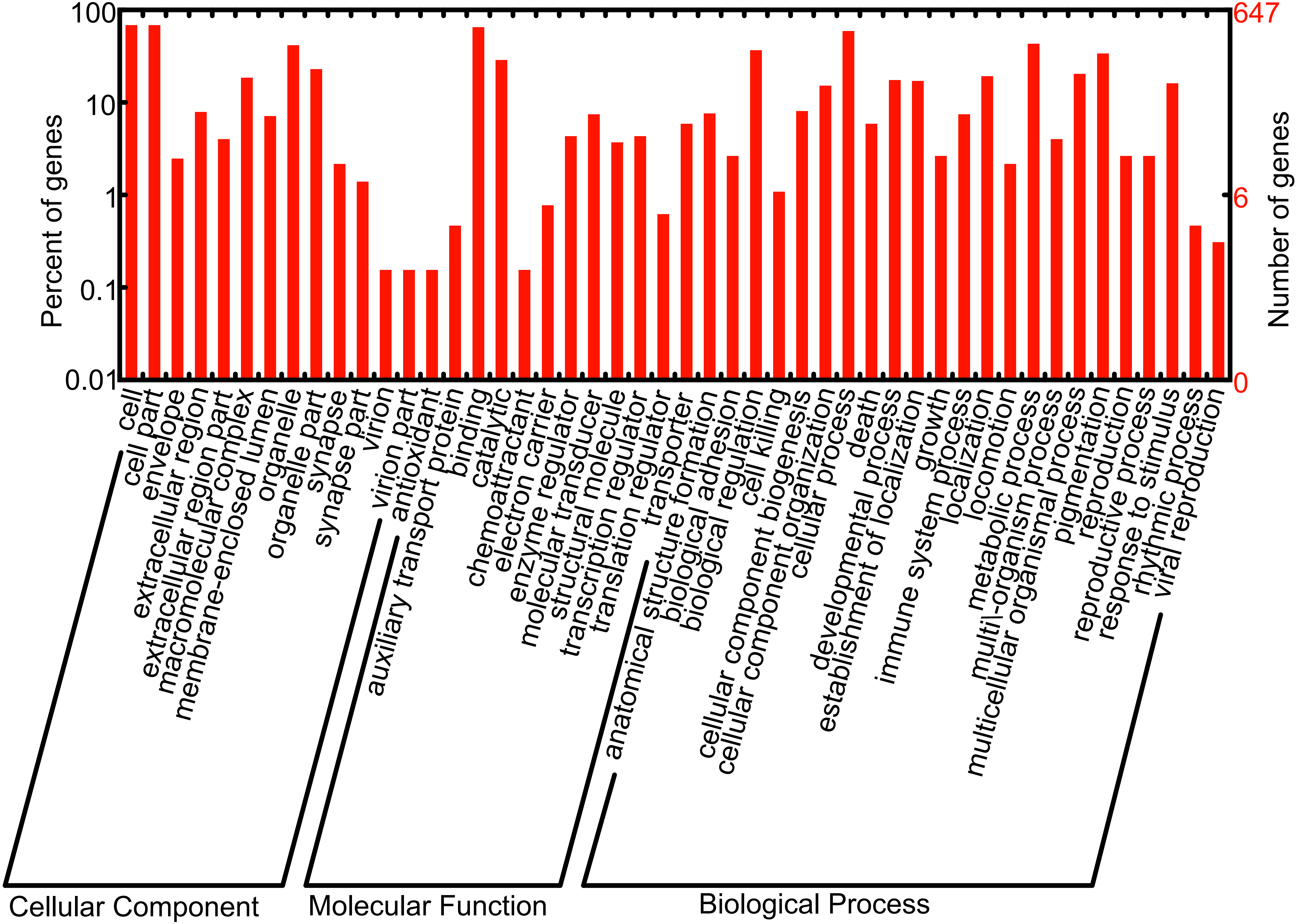

Figure S3 Gene ontology (GO) annotations for genes covered by CNVRs. 647 genes are annotated with GO IDs out of 716 genes covered by CNVRs. Gene annotations with Refgene IDs were done based on genome assemblies of Btau_4.0, Btau_4.6, and Btau_4.6.1, and GO IDs were retrieved in R package biomaRt.

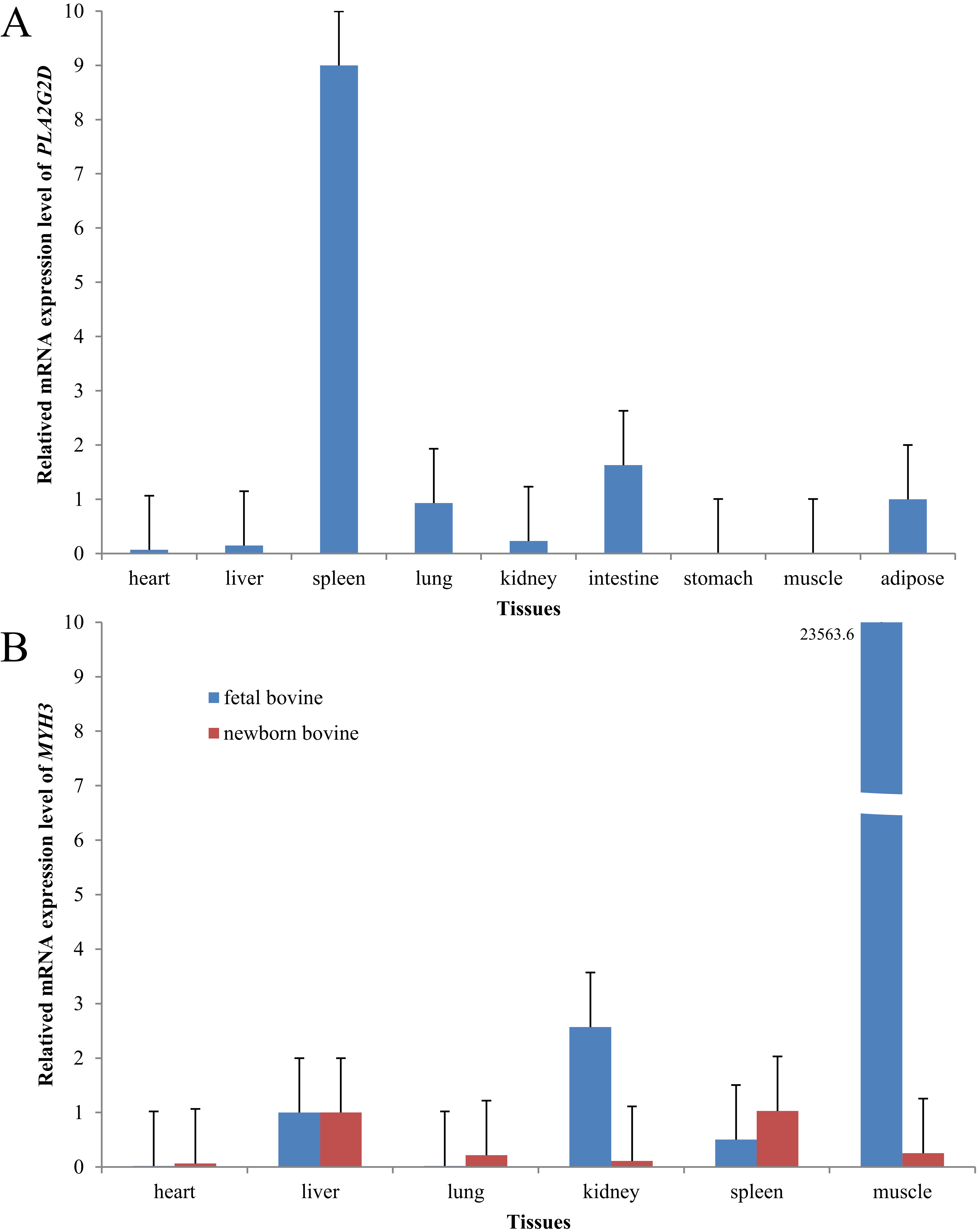

Figure S4 Expression pattern analysis of *PLA2G2D* gene and *MYH3* gene.

A. Expression pattern analysis of *PLA2G2D* gene in several tissues of adult bovine.
B. Expression pattern analysis of *MYH3* gene in several tissues of fetal and newborn bovine. The expression in muscle of fetal bovine was discontinued because it was far out of the others' ranges. Relative mRNA expression levels of *PLA2G2D* gene and *MYH3* gene in different tissues were analyzed by qPCR. mRNA expression were normalized against that of *GAPDH* gene. Error bars represent standard error of the mean (SE). Each column value represents the Means±SE of three replicates.
